# FGFR2 is required for airway basal cell self-renewal and terminal differentiation

**DOI:** 10.1242/dev.135681

**Published:** 2017-05-01

**Authors:** Gayan I. Balasooriya, Maja Goschorska, Eugenia Piddini, Emma L. Rawlins

**Affiliations:** 1 Wellcome Trust/CRUK Gurdon Institute, University of Cambridge, Cambridge, CB2 1QN, UK; 2 School of Cellular and Molecular Medicine, Faculty of Biomedical Sciences, University of Bristol, Biomedical Sciences Building, University Walk, Bristol, BS8 1TD, UK; 3 Wellcome Trust/MRC Stem Cell Institute andDepartment of Pathology, University of Cambridge, Cambridge, CB2 1QN, UK

**Keywords:** Cre-Lox, Lung, Mouse, Trachea, Progenitor

## Abstract

Airway stem cells slowly self-renew and produce differentiated progeny to maintain homeostasis throughout the lifespan of an individual. Mutations in the molecular regulators of these processes may drive cancer or degenerative disease, but are also potential therapeutic targets. Conditionally deleting one copy of FGF receptor 2 (FGFR2) in adult mouse airway basal cells results in self-renewal and differentiation phenotypes. We show that FGFR2 signalling correlates with maintenance of expression of a key transcription factor for basal cell self-renewal and differentiation: SOX2. This heterozygous phenotype illustrates that subtle changes in receptor tyrosine kinase signalling can have significant effects, perhaps providing an explanation for the numerous changes seen in cancer.

## Abstract (precis)

**Summary:** During adult airway epithelial homeostasis in mice, FGFR2 signalling is required for self-renewing divisions of basal stem cells and to maintain expression of the key transcription factor SOX2.

## INTRODUCTION

Like human airways, the mouse trachea contains three major epithelial lineages (Rock et al., 2010; Teixeira et al., 2013). Basal cells (BCs) are a stem cell population and include slowly dividing stem cells and committed luminal precursors (Mori et al., 2015; Rock et al., 2009; Watson et al., 2015). Luminal secretory cells self-renew and produce terminally differentiated ciliated cells (Rawlins and Hogan, 2008; Rawlins et al., 2007, 2009). Multiple studies have shown that SOX2 is a key transcription factor (TF) for the development and maintenance of all airway epithelial cells (Gontan et al., 2008; Hashimoto et al., 2012; Ochieng et al., 2014; Que et al., 2009; Tompkins et al., 2009, 2011). Deletion of *Sox2* in adult mouse tracheal epithelium caused loss of differentiated cells. Moreover, the *Sox2^Δ/Δ^* BCs were less able to proliferate *in vitro* or *in vivo* following injury (Que et al., 2009). SOX2 is thus required for BC self-renewal and luminal differentiation. SOX2 overexpression can be a driver of squamous cell carcinoma, which has a predominantly basal cell phenotype (Correia et al., 2017; Ferone et al., 2016).

FGFR2 function has been extensively studied during lung branching where one of its roles is to maintain undifferentiated epithelial progenitors by inhibiting SOX2 expression (Abler et al., 2009; Que et al., 2007; Volckaert et al., 2013). However, at later stages of embryonic development ectopic FGF10 can promote BC differentiation in SOX2^+^ airway progenitors (Volckaert et al., 2013). The same study expressed a secreted dominant-negative FGFR2 in the late stages of embryogenesis and suggested that there could be a role for FGFR2 signalling in maintenance of airway BCs. We have now specifically tested this hypothesis in the steady-state adult mouse trachea, and show that FGFR2 is required for BC self-renewal and terminal differentiation. Moreover, FGFR2 signalling maintains SOX2 expression.

## RESULTS AND DISCUSSION

### FGFR2 is required for normal tracheal homeostasis

**Fig. 1.**
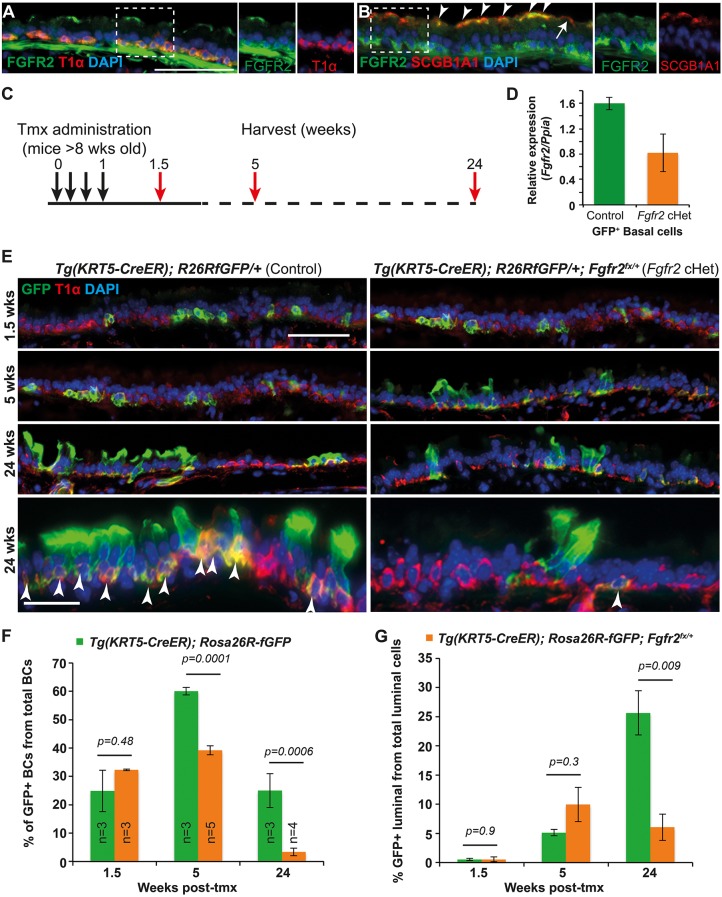
**Decreasing *Fgfr2* levels in basal cells results in altered tracheal homeostasis.** (A,B) Adult tracheal sections. (A) Green, FGFR2; red, T1α (basal cells). (B) Green, FGFR2; red, SCGB1A1 (secretory cells). FGFR2^+^ secretory cells (arrowheads); rare SCGB1A1^+^, FGFR2^−^ cells (arrow). (C) Experimental schematic. (D) Relative expression of *Fgfr2* mRNA in GFP^+^ basal cells from control and *Fgfr2*cHet mice 3 weeks post-tmx. (E) Representative sections from control *Tg(KRT5-CreER); Rosa26R^fGFP/+^* and cHet *Tg(KRT5-CreER); Rosa26R^fGFP/+^; Fgfr2^fx/+^* tracheae. Green, GFP (*Rosa* reporter); red, T1α (basal cells). Arrowheads indicate GFP^+^ basal cells. (F,G) Percentage of the total T1α^+^ BCs that are also GFP^+^ (F) and percentage of the total T1α^−^ luminal cells that are also GFP^+^ (G). Blue, DAPI. Error bars indicate s.e.m. Scale bars: 50 μm.

We detected FGFR2 protein in airway basal cells and at the apical surface of secretory cells (Fig. 1A,B), confirming previous results (Watson et al., 2015). To determine the role of FGFR2 in BCs, we conditionally deleted one copy of *Fgfr2* and activated a GFP reporter in adult tracheal BCs using *Tg(KRT5-CreER); Rosa26R^fGFP/+^; Fgfr2^fx/+^* (*Fgfr2* conditional heterozygous, cHet) and control *Tg(KRT5-CreER); Rosa26R^fGFP/+^* mice (Fig. 1C). To test for co-recombination between *Fgfr2^fx^* and the reporter, we isolated GFP^+^ BCs by flow cytometry as GFP^+^, GSIβ4-lectin^+^ cells at 3 weeks post-tamoxifen (tmx) induction and performed RT-qPCR for *Fgfr2* (Fig. 1D). This confirmed that cHet BCs had ∼50% of the control *Fgfr2* mRNA level. Hence, we use GFP^+^ cells as a surrogate marker for *Fgfr2^Δ/+^* cells, being aware that co-recombination will not be 100%. Tracheae were harvested at intervals to assess the contribution of GFP^+^, *Fgfr2^Δ/+^* BCs to the epithelium during homeostatic turnover (Fig. 1E). At 1.5 weeks post-tmx, ∼30% of total BCs were GFP^+^ in *Fgfr2*cHet and control mice. In controls, this percentage increased to ∼60% at 5 weeks post-tmx, before dropping to initial levels by 24 weeks. By contrast, in the *Fgfr2*cHet tracheae, the percentage of GFP^+^ BCs remained approximately constant at 5 weeks, but decreased to less than 5% of total basal cells by 24 weeks (Fig. 1F). In both genotypes, labelled BCs produced labelled luminal cells. Luminal differentiation initially appeared more rapid in the *Fgfr2*cHets. However, luminal cell production was not sustained over time, likely due to the loss of GFP^+^ BCs, and by 24 weeks the percentage of labelled luminal cells was significantly lower in the *Fgfr2*cHet tracheae (Fig. 1G).

This showed that *Fgfr2*cHet BCs can produce luminal cells, but that mutant basal and luminal cells are gradually lost. One possible reason for the loss of *Fgfr2*cHet cells is differential fitness and competition with neighbouring wild-type cells (Vivarelli et al., 2012). To test this, we mixed pure populations of *Rosa26R^tdTomato/+^; Fgfr2^Δ/+^* with unlabelled *Fgfr2^+/+^* BCs (1:2 ratio) and assessed their ability to compete *in vitro* at steady-state and following injury. We were unable to find evidence for differential proliferation or survival in the mixed cultures and conclude that it is unlikely that cell competition contributes to the observed loss of mutant cells (Fig. S1; Movies 1-5).

### *Fgfr2*cHet BCs do not differentiate into fully mature luminal cells

We asked whether the loss of *Fgfr2*cHet cells was due to a decrease in cell division. As expected, proliferation rates were low in all tracheae, but dividing GFP^+^ cells were observed (Fig. S2A). We noted an increase in proliferation of the *Fgfr2*cHet GFP^+^ cells at 1.5 weeks post-tmx, although this was not statistically significant and the change was not sustained over time (Fig. S2B). Thus, altered proliferation does not explain the phenotype. We also assessed apoptosis using cleaved caspase 3 staining, but did not identify caspase 3^+^ cells (665 GFP^+^ cells scored in four independent 5 week samples; Fig. S2C,D).

**Fig. 2.**
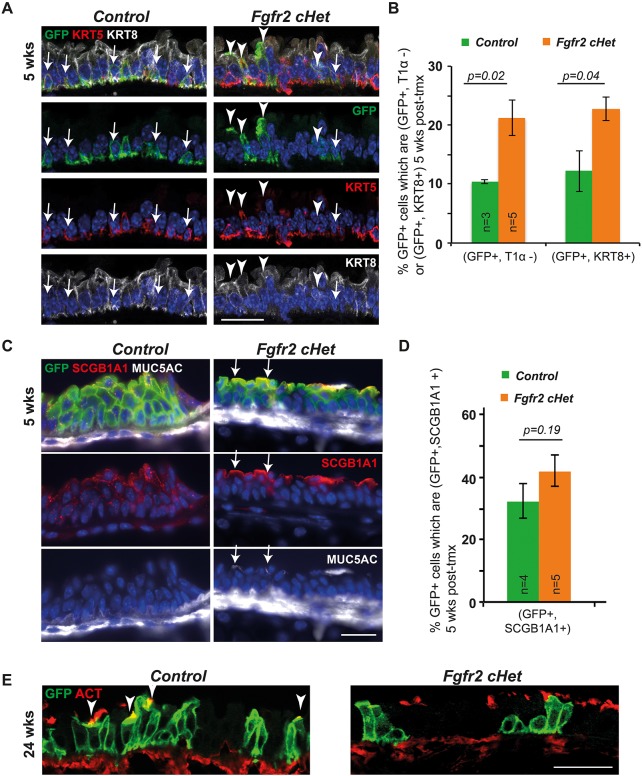
***Fgfr2* conditional heterozygous basal cells do not produce terminally differentiated luminal cells.** (A) Confocal projections from control and *Fgfr2*cHet tracheae 5 weeks post-tmx. Green, GFP (*Rosa* reporter); red, KRT5 (basal cells); white, KRT8 (luminal cells); blue, DAPI (nuclei). Arrowheads indicate GFP^+^ luminal cells. Arrows indicate GFP^+^ basal cells. (B) Percentage of all GFP^+^ cells 5 weeks post-tmx that are GFP^+^, T1 α^−^ (see Fig. 1D) or GFP^+^, KRT8^+^ (see A). (C) Sections from control and *Fgfr2*cHet tracheae 5 weeks post-tmx. Green, GFP (*Rosa* reporter); red, SCGB1A1 (club cells); white, MUC5AC (mucous). Arrows indicate club cells containing a low level of MUC5AC protein. (D) Percentage of all GFP^+^ cells 5 weeks post-tmx that are GFP^+^, SCGB1A1^+^. (E) Confocal sections from control and *Fgfr2* cHet tracheae at 24 weeks post-tmx. Green, GFP (*Rosa* reporter); red, acetylated tubulin (cilia). Error bars indicate s.e.m. Scale bars: 20 μm in A,C; 25 μm in E.

We assessed the ability of *Fgfr2*cHet cells to differentiate by analysing the luminal (KRT8) and basal (KRT5) cytokeratins at 5 weeks post-tmx (Fig. 2A). A higher percentage of the total GFP^+^ cells co-stained with KRT8 in the mutants, indicating that more cells had begun differentiation to a luminal fate (Fig. 2B). Similarly, plotting the GFP/T1α staining (Fig. 1D) as a percentage of GFP^+^ cells (GFP^+^, T1α^−^) showed more differentiating cells in the mutants (Fig. 2B). Thus, *Fgfr2*cHet cells exit the basal layer at a greater rate than controls and their descendants take on a luminal KRT8^+^, T1α^−^ fate, suggesting a self-renewal defect.

At steady-state, BCs initially differentiate into secretory cells that later produce ciliated cells (Watson et al., 2015). Cell fate analysis at 5 weeks post-tmx showed that both control and *Fgfr2*cHet BCs produce secretory SCGB1A1^+^ cells (Fig. 2C,D). Moreover, there were no signs of goblet cell production in the mutants (Fig. 2C; *n*=4 MUC5AC^lo^ cells observed from 859 cells counted in 5 *Fgfr2*cHet individuals). However, analysis of acetylated tubulin-positive cilia (marker of terminal luminal differentiation) at 24 weeks post-tmx showed that the *Fgfr2*cHet cells never took on a ciliated cell identity (Fig. 2E).

### *Fgfr2*cHet BCs have high levels of β-galactosidase activity *in vitro*

**Fig. 3.**
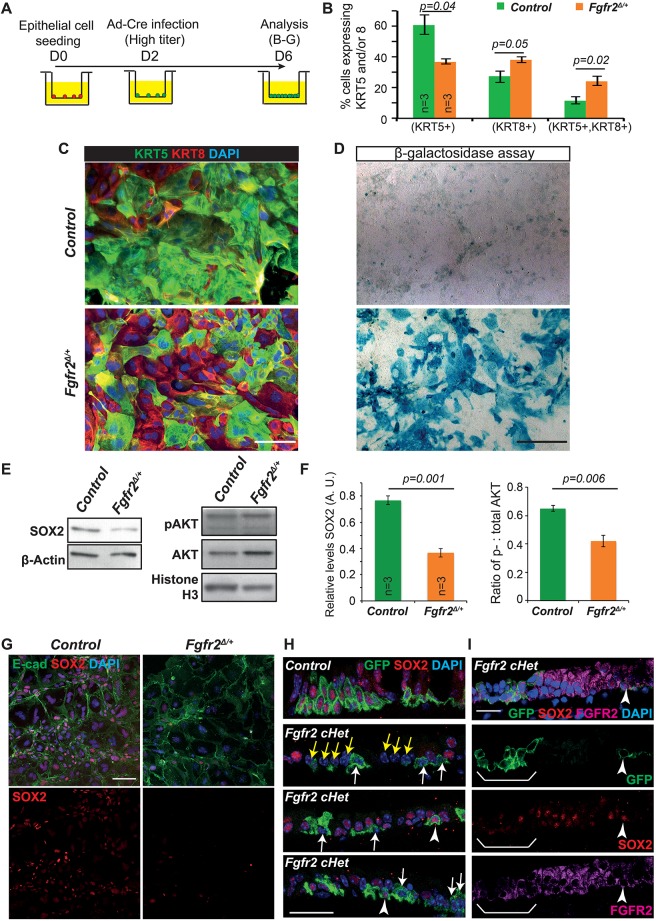
***Fgfr2* conditional heterozygous basal cells have high levels of β-galactosidase and low levels of SOX2.** (A) Experimental schematic for B-G. (B) Percentage tracheal epithelial cells at day 6 post-seeding expressing KRT5 and/or KRT8. (C,D) Control and *Fgfr2*cHet tracheal cells day 6 post-seeding. (C) Green, KRT5 (basal cells); red, KRT8 (luminal cells). (D) X-gal assay for β-galactosidase activity (blue pigment). (E) Representative western blots from control and *Fgfr2*cHet BCs. (F) Quantification of protein levels in E. (G) SOX2 in cHet BCs day 6 post-seeding. Green, E-cadherin (lateral cell membranes); red, SOX2. (H,I) Confocal images of control and *Fgfr2*cHet tracheal sections 5 weeks post-tmx. Green, GFP (*Rosa* reporter); red, SOX2; magenta, FGFR2. White arrows indicate lineage-labelled cells with decreased levels of SOX2. Arrowheads indicate lineage-labelled cells with no change in SOX2. Yellow arrows indicate unlabelled cells with decreased SOX2. Brackets in I indicate a patch of GFP^+^ cells that have decreased FGFR2 and no SOX2. Blue, DAPI. Error bars indicate s.e.m. Scale bars: 100 μm in C; 250 μm in D; 50 μm in G; 25 μm in H,I.

We tested the ability of *Fgfr2*cHet cells to proliferate and differentiate *in vitro* using a high dose of an adenovirus containing CMV-Cre (Ad-Cre) to recombine *Rosa26R^fGFP/+^*; *Fgfr2^fx/+^* and control *Rosa26R^fGFP/fGFP^* BCs grown in self-renewing conditions (Fig. 3A). When analysed by genomic PCR, this resulted in an almost-pure population of *Fgfr2^Δ/+^* cells (Fig. S3A,B). Four days after Ad-Cre-mediated deletion, we observed an increased proportion of KRT8^+^ cells in the *Fgfr2*cHet cultures (Fig. 3A-C). This recapitulates the *in vivo* phenotype and supports the conclusion that *Fgfr2*cHet BCs have a self-renewal defect. Additional cultures were passaged and grown to confluence before differentiation at air-liquid interface (Fig. S4A-D). The *Fgfr2*cHet cells survived passaging but did not reach confluence and failed to express markers of ciliated or basal cell differentiation. Moreover, passaged cells were unable to grow in sphere-forming assays (Fig. S4E-H). The passaged *Fgfr2*cHet cells were somewhat enlarged and flattened, possibly indicating a senescent phenotype (Rodier and Campisi, 2011). We therefore tested for senescence-associated β-galactosidase activity in primary cultures of *Fgfr2*cHet cells. β-Galactosidase activity was detected in 3/3 *Fgfr2*cHet cultures and 0/3 controls (Fig. 3D). Senescence of the *Fgfr2*cHet cells *in vivo* could potentially explain why the luminal GFP^+^ cells can express secretory markers, but do not later produce ciliated cells. However, we cannot absolutely exclude a luminal fate choice defect in *Fgfr2*cHet BCs.

### Lower levels of SOX2 expression in the *Fgfr2* conditional heterozygous cells

We determined the effects of decreasing FGFR2 signalling on downstream pathways using immunoblotting. There was a 1.5-fold decrease in phosphorylated AKT in the *Fgfr2^Δ/+^* cells (Fig. 3E,F), but no change in phosphorylated ERK1/2 (Fig. S3C,D). These changes are consistent with a decrease in FGFR2 signalling via the PI3K-AKT pathway, which was implicated as the main pathway downstream of FGFR2 in adult small airway secretory cells and the developing trachea (Volckaert et al., 2011, 2013).

Most strikingly, there was a twofold decrease in SOX2 in the *Fgfr2^Δ/+^* cells (Fig. 3E,F; Fig. S3C,D). We confirmed the decrease in SOX2 protein at a cellular level by *in vitro* immunostaining (Fig. 3G). Similarly, there was consistently lower SOX2 expression in GFP^+^ cells in the *Fgfr2*cHet tracheae *in vivo* (Fig. 3H, arrows). As expected from the genetic strategy, in the mutants we also observed GFP^+^, SOX2^+^ cells (Fig. 3H, arrowheads) and GFP^−^, SOX2^−^ cells (Fig. 3H, yellow arrows), both are likely to have recombined only one floxed allele. Co-immunostaining with FGFR2 confirmed that the GFP^+^, SOX2^+^ cells observed in the mutants retained high levels of FGFR2 protein (Fig. 3I).

### FGF7 and FGF10 can promote BC colony expansion *in vitro*

**Fig. 4.**
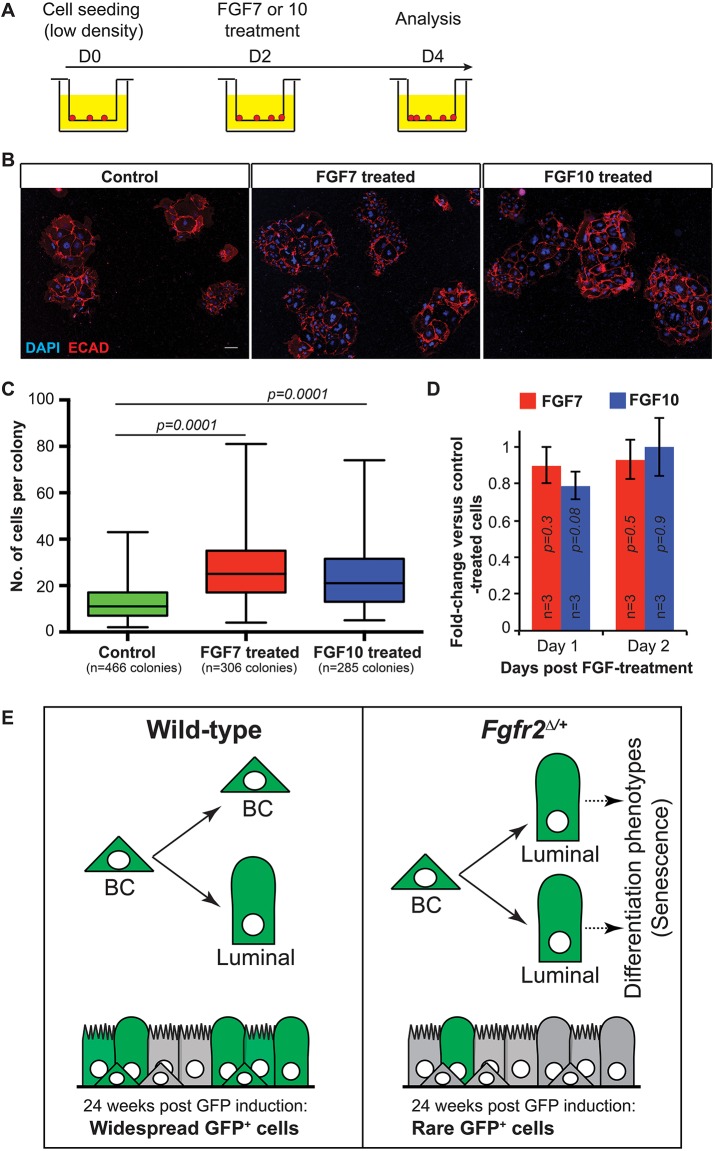
**FGF7 and FGF10 increase colony size of wild-type basal cells.** (A) Experimental schematic. Epithelial cells plated at low density, 3×10^4^ cells/insert. (B) Colonies formed by control, FGF7- or FGF10-treated wild-type cells. Red, E-cadherin; blue, DAPI. Scale bar: 100 μm. (C) Number of cells per colony in B. Data are mean±s.e.m. (D) Level of *Sox2* mRNA relative to control (normalized to 1) in cells treated with FGF7 or FGF10 for 1 or 2 days. Error bars indicate s.e.m. (E) *Fgfr2*cHet BCs rarely make self-renewing divisions in which a new BC is produced. Mutant BCs are more likely to produce descendants with luminal morphology/markers that are unable to completely differentiate, possibly because they senesce. The result is that GFP^+^ *Fgfr2*cHet cells are gradually diluted out from both the basal and luminal populations, and the epithelium is sustained by GFP^−^ wild-type BCs.

We predicted that if a decrease in *Fgfr2* results in loss of BC self-renewal, then activation of FGFR2 *in vitro* should promote the growth of BC colonies. FGF7 and FGF10 are expressed in homeostatic tracheae (Balasooriya et al., 2016) and are known to activate FGFR2 preferentially *in vitro* and *in vivo* (Ornitz et al., 1996). We plated wild-type BCs at low density and added FGF7 or FGF10 on culture day 2 after colonies were established (Fig. 4A). Addition of FGF7 or FGF10 had the opposite effect to decreasing *Fgfr2* and significantly increased colony size (Fig. 4B,C). Interestingly, FGF7 and FGF10 had no effect on the level of *Sox2* mRNA (Fig. 4D).

In conclusion, our data suggest that a normal function of FGFR2 signalling in adult airway BCs is to promote asymmetric self-renewing divisions (Fig. 4E). This is consistent with work in the embryonic trachea where ectopic FGF10 was observed to promote BC fate (Volckaert et al., 2013). By contrast, our previous work on FGFR1 in adult BCs showed that FGFR1 is required to inhibit steady-state proliferation and does not change the ability of BCs to self-renew (Balasooriya et al., 2016). Thus, FGFR1 and FGFR2 have independent functions in airway BCs. We cannot exclude the possibility that they also have other overlapping functions.

We also show that steady-state FGFR2 signalling is required, directly or indirectly, to maintain SOX2 protein levels in the adult airway. This is in contrast to the branching lung, where FGFR2 inhibits SOX2 expression at the tips. Interestingly, an FGFR2-SOX2 inductive relationship has been observed in other cell types (Mansukhani et al., 2005). An FGFR2-SOX2 relationship may be maintained in some squamous lung cancers where *FGFR2* and *SOX2* transcript levels are often correlated (Kim et al., 2016).

### Haploinsufficiency of *Fgfr2* in conditionally deleted adult cells

We were surprised that our *Fgfr2*cHet BCs displayed striking phenotypes when germline *Fgfr2^Δ/+^* animals are viable and fertile (Yu et al., 2003). We therefore looked for subtle epithelial defects in germline-deleted *Fgfr2^Δ/+^* tracheae compared with wild-type siblings, but were unable to find any abnormalities (Fig. S5). *Fgfr2* is haploinsufficient in several organs, including the lacrimal and salivary glands (Shams et al., 2007). We suggest that in mouse embryos heterozygous for *Fgfr2*, genetic compensation operates in most tissues. However, conditional heterozygous deletion in the adult by-passes such mechanisms. This is very similar to recent findings from zebrafish genetics where genetic compensation has been found to operate in germline mutants, but not in acute knockdowns (Rossi et al., 2015). It raises the possibility that many genes that the mouse developmental community assume are uninteresting/redundant based on lack of germline knockout phenotypes do play important roles in development/homeostasis.

## MATERIALS AND METHODS

### Mice

Experiments were approved by local ethical review committees and conducted according to UK Home Office project licenses PPL80/2326 and 70/812. *Fgfr2^fx^* (Yu et al., 2003), *Tg(KRT5-CreER)* (Rock et al., 2009), *Rosa26R-fGFP* (Rawlins et al., 2009), Gt(ROSA)26Sor^tm1(CAG-tdTomato*,-EGFP*)Ees^ (Prigge et al., 2013) and *Fgfr2^Δ/+^* animals were generated by crossing *Fgfr2^fx^* to *Zp3-Cre* (de Vries et al., 2000). The genetic background was C57Bl/6J. Males and females >8 weeks old were used. The wild types were C57Bl/6J.

### Tamoxifen

Adult (>8 week) animals were injected intraperitoneally four times, every other day, with 0.2 mg/g body weight tamoxifen.

### Tracheal epithelial cell culture

Tracheal cells were isolated following published methods (Rock et al., 2009). Briefly, cells were incubated in Dispase II (Gibco, 16 U/ml) for 20 min at room temperature. Epithelial sheets were dissociated using 0.1% trypsin/EDTA. Unless otherwise stated, 5×10^4^ cells in 0.5 ml MTEC/+ media (You et al., 2002) were plated on collagen-coated 12-well tissue culture inserts (BD Falcon, 353180). For tracheospheres, cells were passaged into 50% matrigel (Becton Dickinson). Adeno-Cre (University of Iowa, Gene Transfer Vector Core) was incubated at MOI 2500; vector pfu 1×10^6^ for 8 h. Recombinant mouse FGF7 and FGF10 (R&D Systems) were used at 100 ng/ml. For competition assays, mixed populations of cells were grown to confluence and then imaged every 4 h for 10 days in a Nikon Biostation. Alternatively, confluent cultures were scratched and imaged every 2 h for 5 days. *In vitro* experiments were preformed in triplicate.

### Immunostaining

Tracheae were fixed in 4% paraformaldehyde at 4°C for 4 h; washed PBS, sucrose protected, embedded in OCT (Optimum Cutting Temperature Compound, Tissue Tek) and sectioned at 6 μm. Airway culture inserts were washed in PBS, fixed for 10 min in 4% paraformaldehyde at room temperature and permeabilized with 0.3% Triton X-100. Primary antibodies are listed in Table S1. Alexa Fluor-conjugated secondary antibodies (1:2000) were from Life Technologies (Table S1). DAPI and fluoromount were from Sigma. X-gal staining was performed using Senescence β-galactosidase staining kit (Cell Signaling, 9860).

### Microscopy and image scoring

Slides were imaged on a Zeiss AxioImager compound, or a Leica Sp8/Sp5 confocal microscope. Cells were scored manually in Fiji. For cryosections, every epithelial cell along the entire proximal to distal length of a longitudinal section from the centre of the trachea was scored. For cultured cells at least three random fields of view from each insert were scored. Raw cell counts are available in Fig. S6.

### RT-qPCR

Primary tracheal epithelial cells were isolated and sorted using a fluorescence-activated cell sorting MoFlo flow cytometer. GFP^+^ basal cells from control and *Fgfr2*cHet tracheae were sorted as GFP^+^, GSIβ4 lectin^+^ (Balasooriya et al., 2016). Total RNA was extracted using Qiagen RNEasy Mini Kit. Taqman gene expression assays for *Ppia* (Mm02342429_g1), *Fgfr2* (Mm01269930_m1) and *Sox2* (Mm03053810_s1) (Life Technologies) were used.

### Immunoblots

Cells were collected in Cell Extraction Buffer (Invitrogen, FNN0011) with protease inhibitor (Roche 04693116001) and PMSF (Sigma, P7626). Proteins were separated on 10% or 12% SDS-PAGE gels before being transfer onto Millipore Immobilon-P PVDF Membrane (Merck Millipore, IPVH00010). Primary antibodies are listed in Table S1. Detection with HRP-conjugated secondaries (Abcam, 1:10,000) and enhanced chemiluminescense (Thermo Scientific, PI-32109) was carried out. Quantitation is based on protein from three biological replicates separated on the same polyacrylamide gel. Band intensity was analysed in Fiji normalised to the loading control.

### Statistics

*P*-values were obtained using an unpaired two-tailed student's *t*-test with unequal variance.
